# Forecasting Milking Efficiency of Dairy Cows Milked in an Automatic Milking System Using the Decision Tree Technique

**DOI:** 10.3390/ani12081040

**Published:** 2022-04-16

**Authors:** Joanna Aerts, Magdalena Kolenda, Dariusz Piwczyński, Beata Sitkowska, Hasan Önder

**Affiliations:** 1 Lely Services B.V., Cornelis van der Lelylaan 1, 3147 PB Maassluis, The Netherlands; jaerts@lely.com; 2 Department of Animal Biotechnology and Genetics, Faculty of Animal Breeding and Biology, Bydgoszcz University of Science and Technology, 85-084 Bydgoszcz, Poland; darekp@pbs.edu.pl (D.P.); beatas@pbs.edu.pl (B.S.); 3 Department of Animal Science, Ondokuz Mayis University, Samsun 55139, Turkey; honder@omu.edu.tr

**Keywords:** milking efficiency, automatic milking system, decision trees, dairy cattle

## Abstract

**Simple Summary:**

Automatic milking systems are gaining popularity worldwide as they help in monitoring milk production traits, inter alia, milking efficiency defined as milk yield divided by box time. In our study we used a statistical method called the decision tree technique, which allows us to study the impact of specific factors on milking efficiency and display them as a simple graphical model. By studying the tree a farmer might identify the factors most affecting milking efficiency.

**Abstract:**

In barns equipped with an automatic milking system, the profitability of production depends primarily on the milking efficiency of a cow (ME; kg/min) defined as cow milk yield per minute of box time. This study was carried out on 1823 Polish Holstein–Friesian cows milked by the automatic milking system (AMS) in 20 herds. Selected milking parameters recorded by the AMS were analyzed in the research. The aim of the study was to forecast ME using two statistical techniques (analysis of variance and decision trees). The results of the analysis of variance showed that the average ME was 1.67 kg/min. ME was associated with: year of AMS operation (being the highest in the first year), number of cows per robot (the highest in robots with 61–75 cows), lactation number (highest for multiparas), season of calving (the highest in spring), age at first calving (>36 months), days in milk (151–250 days) and finally, rear quarter to total milk yield ratio (the highest between 51% and 55%). The decision tree predicted that the highest ME (2.01 kg/min) corresponded with cows that produced more than 45 kg of milk per day, were milked less than four times/day, had a short teatcup attachment time (<7.65 s) and were milked in robots that had an occupancy lower than 56 cows.

## 1. Introduction

During recent decades milk production has changed greatly, due, among other reasons, to the implementation of new milking systems including the automatic milking system (AMS). Automatization in dairy farms has been introduced, for instance, in the form of milking robots, automated milking parlours and rotary milking parlours. The AMS is gaining in popularity in Europe [1,2] due to the possibility of reducing manual labour on dairy farms as the AMS aids farmers with teat-cleaning, attaching teat cups and disinfecting teats after each milking. One of the advantages of the AMS is that the milking frequency per day may increase as cows have access to the AMS regardless of farm employee presence and may use it when needed. This may result in higher milk yield [3,4,5]. It is well known that the profitability of milk production depends heavily on the milking efficiency of a cow. Milking efficiency may be affected by different factors, including: milking frequency (lower contributes to a lower yield), number of cows per robot (a high number of animals may increase competition in the herd and negatively affect yield) or type of barn in which the animals are milked (new barns built specifically for AMS use may be better adjusted to this purpose and therefore allow cows to use the AMS more efficiently, increasing yield) [6,7,8,9,10]. In the AMS milking efficiency is mostly described as milk yield per minute of box time [11]. The highest milking efficiency is obtained with the best ratio between high milk yield from a cow and the short time spent by said cow in a robot [12]. Heringstad and Bugten [13] noted that ME changes with days in milk (DIM). ME is the lowest during early lactation and highest in mid-lactation, after which it decreases. It is also worth noting that ME is affected by inter alia, the herd, season, milk yield, milking frequency and milking speed.

Many authors have pointed out that milking performance of cows is a complex issue affected by many factors such as number of cows per robot, robots per barn, box time, number of connection attempts or type of traffic (free/forced) [9,10]. ME is an important factor that affects profitability of production and therefore, studies aiming at forecasting ME using prediction models may be beneficial. One of the statistical methods used to forecast the milking efficiency is a decision tree technique, which helps visualise data in a simple tree-like graphical way. This technique creates structures that resemble trunk, branches and leaves. Division of the datasets starts with a root node that is further divided into child nodes, which may divide subsequently until no further division can be made thus creating a leaf. Once the tree is developed it consists of subsets that are maximally homogeneous in terms of the value of the tested traits. Such tree allows one to identify the factors and their levels that contribute to the highest and the lowest values of the tested trait [14]. The authors of the present study have shown that the decision tree technique may be an alternative to analysis of variance or multiple linear regression [15,16].

The aim of this study was to forecast the milking efficiency of Polish Holstein–Friesian cows using the decision tree technique, and at the same time to determine the sources of variability of milk speed and yield, milking frequency, attachment time and box time in automatic milking systems.

## 2. Material and Methods

### 2.1. Animals

The study included a total of 1823 Polish Holstein–Friesian cows kept in 20 dairy farms located in Poland (Table 1). Cows were milked using an automatic milking system (AMS; Astronaut A4 by Lely Industries N.V.: Cornelis van der Lelylaan 1, Maassluis, The Netherlands) and were fed a partial mixed ration (PMR), with concentrate feed that was given to animals individually in the milking box depending on their milk yields. Data were collected from dairy cows in their first to third lactation. Data on milking performance of cows milked in AMS were obtained from the T4 C management and data registration system by Lely East. A total of 713,206 records were obtained. The following milking performance variables were tested in this study:

- Days in milk (days, DIM)—average number of milking days;
- Milking frequency (number/day, MF)—number of milkings per cow milked by AMS per day;
- Attachment time per milking (s, AT)—the average time per milking per cow that it took the robot to attach the teatcup;
- Box time (min/day, BT)—the total time spent by a cow in the milking box during a day;
- Milk speed (kg/min, MS)—average milk flow rate per cow per day of robot operation;
- Milk yield (kg/day, MY)—total daily milk yield of a cow per day;
- Ratio of rear quarter MY to total (front and rear quarter) MY (%, RTR),
- Milking efficiency (ME, kg/min)—milk yield per day divided by box time.

**Table 1:** Characteristics of studied dairy herds milked with automatic milking system.

Her	Number of Robots per Herd	Mean No. of Cows per Robot	Laying Area	Walking Area
A	1	54	Mats	Grates
B	1	59	Mats	Grates
C	1	66	Mats	Grates
D	2	53	Straw	Grates
E	1	55	Straw	Concrete
F	2	51	Mats	Grates
G	1	56	Mats	Concrete
H	1	65	Mats	Concrete
I	1	59	Mats	Grates
J	3	44	Straw	Grates
K	1	55	Mats	Concrete
L	1	63	Mats	Grates
M	1	58	Mats	Grates
N	5	50	Straw	Concrete
O	1	58	Mats	Grates
P	2	53	Mats	Concrete
R	1	59	Mats	Grates
S	3	52	Mats	Grates
T	1	56	Mats	Grates
W	1	62	Mats	Grates

### 2.2. Statistical Analysis

Other variables that may affect milking efficiency were taken into consideration: year of AMS operation (yAMS: 1, 2 or 3 years), number of cows per one robot (noC: 45–50; 51–55; 56–60 or 61–75 cows), lactation number (noL: 1 or higher (2 or 3)), season of calving (SC: autumn, spring, summer or winter) and age at 1st calving (AFC). For the purposes of statistical analyses, the above-mentioned variables were categorized as follows: DIM: <50, 51–100, 101–150, 151–200, 201–250 and 251–305 days; MF: 1, 2, 3, 4 and ≥5); MY: <25, 25–30, 31–35 and 35–45 kg; RTR: 34–50%; 51–55%; 56–60% and 61–73%.

The statistical analysis of the collected data was performed in three stages. First, using the mixed model repeated measures analysis of variance (procedure mixed SAS) the analysis of milk yield variability and recorded milking parameters was performed [17]. For this purpose, the following mixed model was used (1):

y_ijklmnoprs_ = μ + yAMS_i_ + Barn_j_ + noC_k_ + nL_l_ + SC_m_ + AFC_n_ + DIM_o_ + RTR_p_ + (nL × SC)_lm_ + a_r_ + e_ijklmnoprs_ (1)

where:

y_ijklmnopqrs_—the phenotype value of the trait (ME, MF, AT, BT, MS, MY),
µ—a general average,
yAMS_i_—the fixed effect of the *i*th yAMS class,
Barn_j_—the fixed effect of the *j*th barn type,
noC_k_—the fixed effect of the *k*th class of noC,
nL_l_—the fixed effect of the *l*th noL,
SC_m_—the fixed effect of the *m*th SC,
AFC_n_—the fixed effect of the *n*th AFC class,
DIM_o_—the fixed effect of the *o*th DIM class,
RTR_p_—the fixed effect of the *p*th RTR class,
(nL × SC)_lm_—interaction nL × SC,
a_r_—the random effect of *r*th cows,
e_ijklmnoprs_—random error.

The significance differences between the selected groups were established using the Scheffe test (SAS).

In the next step of the statistical procedure, the Pearson correlation coefficients between ME and MF, AT, BT, MS and MY were calculated. The aim of this task was to reduce the number of variables used in the next stage of analysis for prediction of ME.

Then, in the third stage of statistical analysis—the main stage of research—attempts were made to forecast ME using the decision tree technique [17]. All variables were taken into consideration; however, MS was excluded from the model as a high correlation (0.998) between ME and MS was noted.

In ME forecasting the CART (classification and regression trees) algorithm was ultimately used. This algorithm uses variance reduction as a tree division criterion. Due to the continuous nature of the tested variable (ME), two different division criteria were taken into consideration while creating the decision tree—F test statistic and variance reduction [18]. Subsequently trees constructed based on these criteria were compared in terms of the quality of forecasting of ME based on the average squared error. It is worth mentioning that the decreasing value of the average squared error indicated a higher quality of the model. During construction, mean square error was used as a measure to select the best tree based on validation data. Data were divided into training and validation sets. The “training set”, which was used to preliminarily fit the model, was constituted from 60% of all observations, while the “validation set” (the set that prevented modelling from overfitting, that is, it prevented an excessive fit of the tree model to the data on the basis of which it was created) was constituted from 40% of all observations. Cows were assigned to the training or validation sets by the random sampling method. It was assumed that the minimum size of a leaf should not be less than 5000 observations, and the maximum depth of a tree (number of branches) should not be deeper than 5.

**Figure 1:**
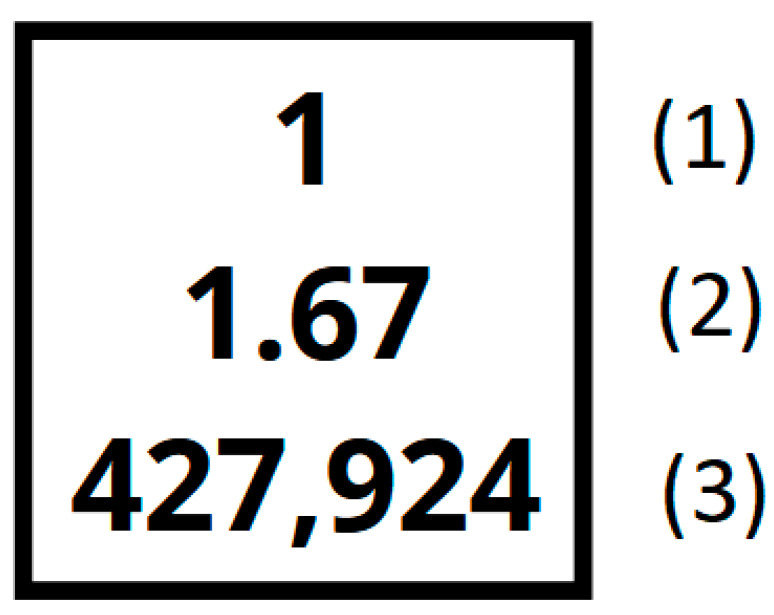
The description of the root node, including the node ID (1), milking efficiency (2) and number of observations in a node or a leaf (3).

Each node of the created tree was composed of the following data: node ID (1); milking efficiency (2); and number of observations in a given node or a leaf (3) (Figure 1). An “Importance” measure was used to create a ranking of different variables in terms of their importance in splitting the dataset [17,19]. The “Importance” measure and the way in which it was calculated are provided in the paper by Grochowska et al. [19]. The “Importance” measure takes values between 0 and 1, and a higher value indicates a greater importance during tree construction. This ranking also provides the data on the number of node divisions that were performed based on respective variables [16,17,18,19].

## 3. Results

### 3.1. Analysis of Milk Yield Variability and Recorded Milking Parameters with the Use of Multivariate Analysis of Variance

Descriptive statistics of analysed traits are presented in Table 2. Cows produced on average of 29 kg of milk per day. While the average box time per milking was 18.41 min, cows spent approximately 6.03 min attached to a robot and milked on the average 2.83 times a day. Results showed that 54.5% of the milk produced by the cows was collected from rear quarters. The results showed that the average milking efficiency in the analyzed population of cows was 1.67 kg/min (Table 2).

The analysis of variance showed a highly significant influence of yAMS, noC, noL, SC, AFC, DIM, RTR and noLx SC on ME, MF, AT, BT, MS and MY. Barn type was not significantly associated with ME, MF, MS or MY.

From the mixed-model analysis it was noted that ME was affected by the following factors: yAMS (being the highest in the first years), noC (the highest in robots with 61–75 cows), noL (highest for multiparas), SC (spring), AFC (>36 month), DIM (201–305 days) and finally by RTR (the highest between 51 and 55). It was also noted that the interaction between noL and SC affected ME (Table 3).

Higher levels of MF, AT, BT and MY were noted in barns that were adapted to AMS use although only for AT and BT were the differences in the levels of features between new and adapted barns significant. The highest MF and the longest BT were found in herds with 51 to 55 cows per robot. The longest AT and highest MS, MY, and ME were recorded in the herds with the highest density (over 60 cows per milking robot). The lowest MY, MS and ME were recorded in the herds with the lowest density per robot (fewer than 51 cows).

Lactations 2 and 3 were associated with higher ME, MF, BT, MS, and MY, and lower AT. Differences between lactations were statistically significant (Table 3).

Cows calving in the autumn and winter were characterized by significantly higher levels of MF, BT and MY compared to summer and spring. The highest values of AT, MS and ME were found in the spring. The lowest levels of MS, MY and ME were observed in summer.

Age at first calving significantly differentiated the level of the examined traits. MF, BT and MY were significantly higher with an AFC of 26–36 months and ME and MS with an AFC of more than 36 months. AT was significantly higher with an AFC of 24–25 months.

When analyzing days in milk, the highest levels of MF, AT, BT and MY were recorded up to the 100th day of lactation, and in subsequent stages the levels decreased. On the other hand, higher levels were noted later in lactation for ME (151–250 days) and MS (201–250 days). It was observed that the higher the share of rear quarter to total quarter MY ratio, the higher the levels of MF, AT, BT, MY and ME. The highest MS was found for milkings whose rear quarter to total quarter MY ratio was between 51 and 55% (Table 3).

Correlation between milking efficiency and other tested traits that characterized milking processes were investigated (Table 4). The results showed the high correlation between ME and MS (0.879), therefore MS was not included in the algorithm creating decision tree model.

### 3.2. Forecasting ME

The study found that the decision tree models built on the basis of the alternative two division criteria—F test statistic and variance reduction—had the same prognostic ability, as evidenced by the same mean standard error (0.18).

A decision tree method is of particular value to people who, not familiar with statistical analysis, would like to verify the influence of various factors on a tested trait solely on the basis of the graphical model [20]. Such people, following the graphical presentation of splits, may identify the factors and their levels that may contribute to improvement of the investigated trait.

**Figure 2:**
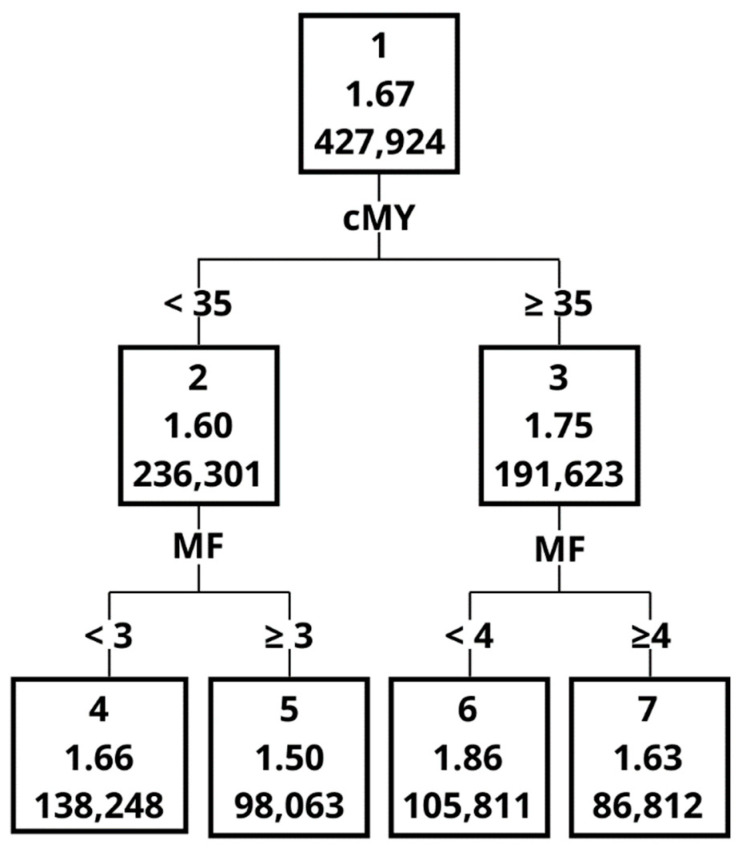
The graphical model of the decision tree—part 1. Abbreviations used in Figure 2, Figure 3, Figure 4, Figure 5 and Figure 6: cMY—milk yield; MF—milking frequency; AT—attachment time; DIM—days in milk; noL—lactation number; noC—number of cows per robot; RTR—rear quarter to total quarter MY ratio; AFC—age at 1st calving; SC—season of calving.

**Figure 3:**
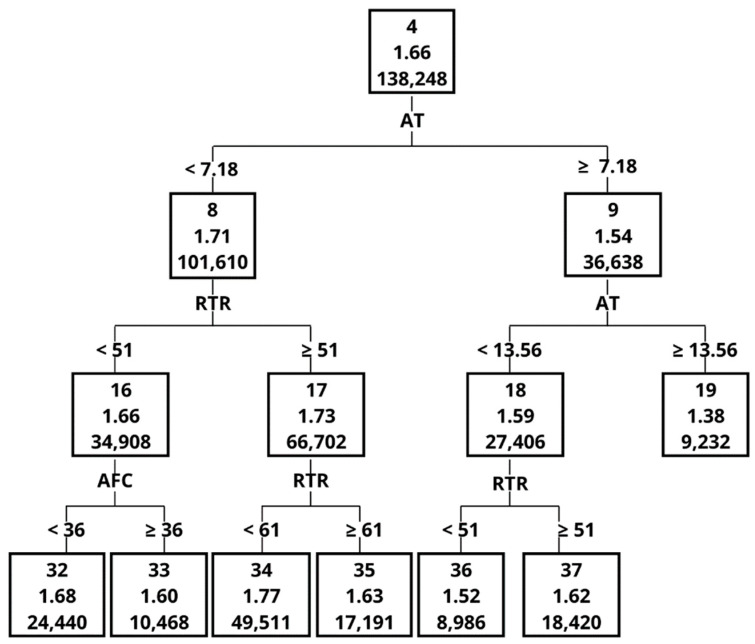
The graphical model of the decision tree—part 2.

**Figure 4:**
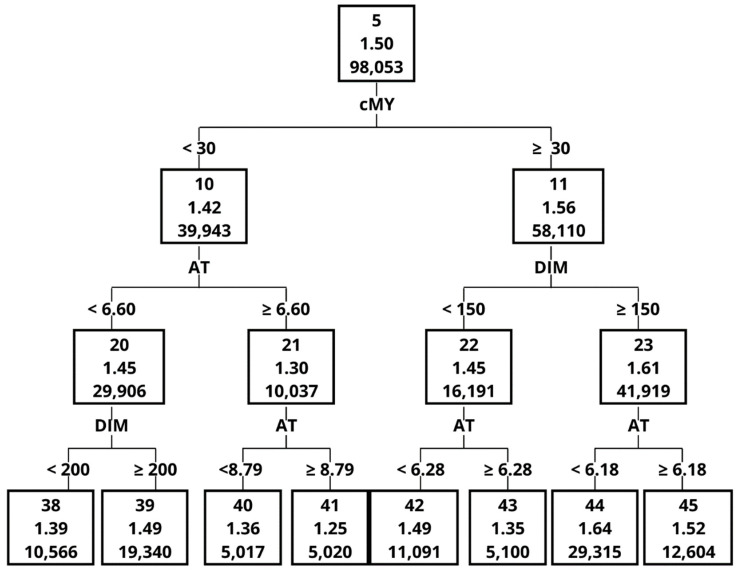
The graphical model of the decision tree—part 3.

**Figure 5:**
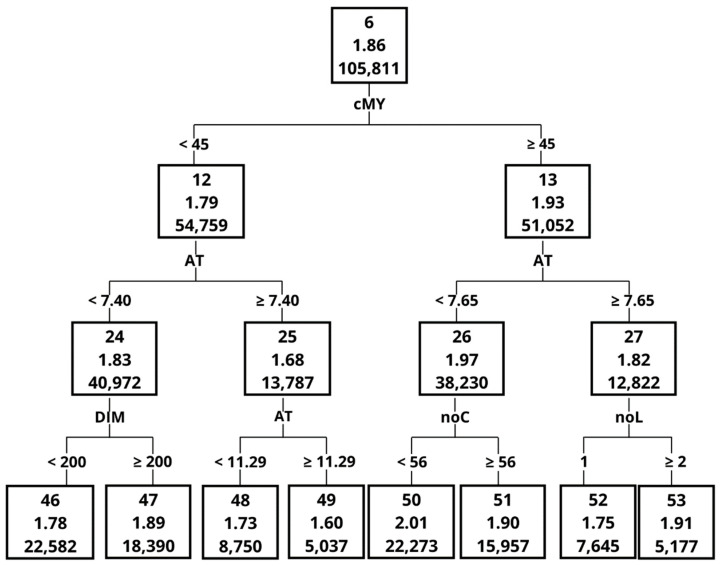
The graphical model of the decision tree—part 4.

**Figure 6:**
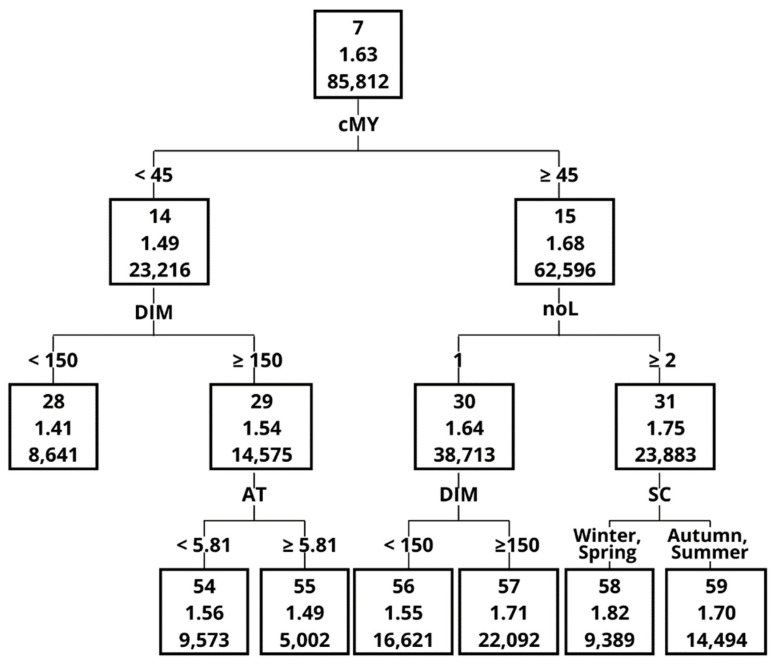
The graphical model of the decision tree—part 5.

The Importance measure, calculated on the basis of the validation set, indicated that the greatest impact on ME in the order of decreasing importance was MY, MF, AT, DIM, noL and noC (Table 5). One of the most important variables, with an Importance measure of 1 was MY, although it contributed only to 4 splits. The graphical representation of the decision tree (Figure 1, Figure 2, Figure 3, Figure 4, Figure 5 and Figure 6) showed that cows that were characterised by a higher MY also had a higher ME. The variable that caused the tree to split the most was the AT (10 splits) and DIM (5 splits).

The decision tree (Figure 2, Figure 3, Figure 4, Figure 5 and Figure 6) was 5 levels deep and resulted in the creation of 59 nodes out of which 29 were leaves. The first division in the graphical model of a decision tree was made based on MY, splitting data with MY below 35 kg (Node 2, the average ME at the level of 1.60 kg/min) and above (Node 3, with ME 1.75 kg/min). Node 3 contained data for which ME had a higher value and was further split by MF with 4 times/day as a threshold creating nodes 6 and 7. The leaf with the highest ME was Node 50 with ME of 2.01 kg/min. The leaf was created by splitting the original dataset by the following criteria: MY (≥35 kg/day), MF (<4 times/day), MY (≥45 kg/day), AT (<7.65 s) and number of cows per robot (<56 cows). In contrast, the leaf (Node 41) with the lowest ME (1.25 kg/min) was created by the following splits: MY (<35 kg/day), MF (≥3 times/day), MY (<30 kg/day), AT (≥6.60 s) and AT (≥8.79 s).

## 4. Discussion

### Milking Efficiency and Parameters Affecting It

On average, ME in the present study was 1.67 kg/min. Lower efficiency was described by other authors, including Vosman et al. [21] who reported it to be at a level of 1.61 kg/min for Holstein–Friesian cows. Heringstad and Bugten [13] also reported lower values of ME (the average daily ME was 1.47 kg per min of box time for Norwegian Red cows). Higher ME in our study may have been caused by the fact that cows selected for this study came from herds that are considered to be one of the best in Poland.

The study predicted the creation of a graphical model with the use of decision tree techniques. These methods split datasets in order to indicate what variables affect ME. The algorithm creating the graphical model of a decision tree gave the highest importance in relation to ME to milk yield. Løvendahl et al. [11] concluded that in AMS a cow that gives the most milk in one minute of box time should be called the “AMS milking efficient cow”.

The decision tree algorithm indicated MF as second-most important variable (with an Importance of 0.984) that affected the tree creation. Castro et al. [9] reported that in their study on Holstein cows milked in an AMS, the optimal MF was between 2.40 and 2.60 milkings/day. In our study the best Node in the graphical presentation of the tree was created by a split where the threshold for MF was below 4; while the average MF for the whole tested population was 2.83 milkings/day. Some authors consider that a minimum accepted MF of a cow should be determined by a technician on the basis of the age of a cow and the lactation stage [5]. MF is a crucial parameter that affects MY as well as ME [7,11,22]. The automatic milking system allows the animals to independently choose the moment of milking almost all day long and may contribute to the increase of the frequency of milking [5,23]. Løvendahl and Chagunda [24] pointed out that cows (Red Dane and Holsteins) with higher MF gave 20% more milk than cows with lower MF.

The highest number of splits was caused by the variable AT, which suggests, along with the fact that the Importance was also high, that it greatly affects ME. Bach and Busto [25] suggested that attachment failures have a great effect on the overall milk yield. Failure to attach the cups to the target teat negatively affect milk ejection in other, unaffected, quarters. Piwczyński et al. [26] suggested that a short AT may improve the profitability of automatic milking. A rapid attachment of a teatcup to a target teat also reduces the stress on a cow as well as the time a cow stays in the milking robot. This also means that a cow frees up the space in the robot for the next animal increasing the number of cows using one robot, which also affects profitability of the farm.

DIM had an importance of 0.678. The tree showed that cows with DIM lower than 150 days had lower ME. This is in accordance with findings of Heringstad and Bugten [13] who reported that ME tended to be low at the beginning of lactation and increased with time, to start decreasing at the end of lactation when MY also decreased.

While the tree was split based on lactation number only twice, the Importance value was moderate. The study showed that higher ME was recorded in second and third lactations. These results are in accordance with those reported by Vosman et al. [21]. They noted that the difference between the average ME in the 1st and 3rd lactation was 0.25 kg/min. Carlström et al. [27] also reported higher values of milk yield per milking, which contributed to the results showing a higher ME in the 2nd lactation (2.58 kg/min) compared to the 1st lactation (2.36 kg/min), and Spolders et al. [28] confirmed that primiparas had a higher milking performance.

The graphical model indicates that the number of cows using the same robot is also very important and may affect ME. To our knowledge the literature does not provide direct information on the relationship between stocking rate and ME (described as milk yield per day divided by box time) in AMS, but other authors confirm that the occupancy rate affects MY, although not all authors confirmed statistical significance [29,30]. It may be concluded that the optimal stocking level per one AMS is crucial since some authors reported the positive effect of an increase in the number of cows per AMS (average occupancy rate at the level of 55.8 cows per AMS) [29] on MY (kg/cow), while others noted a decrease in MY (kg/cow per day) related to the increase of number of cows/robot [30]. Lee et al. [31] reported that the mean MY per AMS went up with an increase in the number of cows per AMS, however, only until it reached the number of 60 cows per robot; further increase of the stocking level did not improve MY. This suggests that while initial increasing of the number of cows using AMS may reduce the robot free-time, thus contributing to the increase of MY, the number of cows per robot cannot be increased indefinitely, as the maximum robot throughput will eventually be reached. Further increasing the occupancy rate will not improve MY, and on the contrary, may decrease MY due to increasing competition between cows.

RTR was used to split the tree three times. It is well known that different udder quarters have a different time of milking. The difference is especially seen between front and rear quarters [32]. Sitkowska et al. [32] pointed out that in their studies rear quarters took 43 s longer to be milked than front quarters. They also reported the total MY (10.65 kg) as MY per front (4.80 kg) and rear (5.85 kg) quarters. This allowed them to calculate RTR (55.02%) which is higher than that reported in the present study (54.49%). Nevertheless the importance (0.259) of this variable is not very high in tree creation, which suggests that it does not significantly affects ME.

AFC was used only once during the creation of the tree and the importance of that variable was low (0.131) suggesting that it did not affect ME. In Poland in 2020 the average AFC was 812 days [33]. The optimal age of the first calving determines the proper development of the udder and adaptation to automatic milking and may contribute to the reduction of reproductive and health-related disorders. Attempts have been made to indicate the optimal AFC at which a cow reaches its best production potential [34,35]. Nilforooshan and Edriss [36] indicated that for Iranian Holsteins the optimal AFC was 24 months. In our study analysis of variance indicated that the highest ME had cows whose AFC was higher than 36 months.

Also SC had a low Importance in tree creation and did not significantly affect ME; however, analysis of variance indicated that SC had a significant impact on ME (with the highest ME in the spring season and the lowest in summer). Speroni et al. [37] also reported that a hot season had a negative effect on MY in AMS (−4.5 ± 0.6 kg/d).

One of the main goals of a farmer running a dairy farm is a high level of profitability of their herd. Therefore, they are constantly endeavouring to increase the efficiency of milking in AMS. Vosman et al. [21] pointed out that to do so, farmers should breed only those cows that are suitable for AMS (active in the robot, visit it frequently, have high milk yield but at the same time have a relatively low box time). Løvendahl et al. [11] indicated that ME expressed a higher heritability than residual BT, and that it was also highly correlated to other investigated traits, therefore, it may be used in a selection programme aimed at obtaining cows with a high ME in AMS.

## 5. Conclusions

The decision tree model, in an easily comprehended graph, indicated the best combination of factors and their levels that contributed to the highest ME in herds milked in AMS. The results showed that the highest ME was recorded for cows that produced more than 35 kg per day, were milked in robots that had occupancy lower than 56 cows and were kept in new barns where AMS was installed at least 3 years prior. This research may be beneficial for farmers as it depicts, in a simple way, how different factors may contribute to the increase of the efficiency of their milk production.

## Figures and Tables

**Table 2:** Descriptive statistics of tested traits.

Variable	N	Mean	SD	CV (%)
Number of cows per robot (n)	713,206	55.37	8.36	15.09
Age at 1st calving (days)	713,206	907.30	237.26	26.15
Days in milk (days)	713,206	149.65	84.22	56.28
Milking frequency (n/day)	713,206	2.83	0.91	31.95
Attachment time (s)	713,206	6.03	3.92	65.05
Box time (min/day)	713,206	18.41	7.57	41.12
Milk speed (kg/min)	713,206	2.59	0.91	34.93
Milk yield (kg/day)	713,206	29.03	9.92	34.17
Rear quarter to total quarter MY ratio (%)	658,159	54.49	7.02	12.88
Milking efficiency (kg/min)	713,206	1.67	0.46	27.33

SD—standard deviation, CV—coefficient of variation.

**Table 3:** Impact of selected factors on tested traits (least square means).

Factor	Level	ME	MF	AT	BT	MS	MY
Year of AMS operation	1	1.71 ^A^	2.93 ^A^	5.91 ^A^	18.74 ^A^	2.67 ^A^	30.30 ^A^
	2	1.71 ^B^	2.88 ^AB^	6.08 ^AB^	18.65 ^AB^	2.65 ^AB^	30.29 ^B^
	3	1.69 ^AB^	2.77 ^AB^	6.00 ^AB^	18.36 ^AB^	2.59 ^AB^	29.56 ^AB^
Barn type	Adapted	1.69	2.88	6.15 ^A^	18.98 ^A^	2.63	30.34
	New	1.72	2.84	5.82 ^A^	18.19 ^A^	2.65	29.76
Number of cows per robot	45–50	1.65 ^A^	2.89 ^A^	5.95 ^Aa^	18.62 ^A^	2.59 ^A^	29.26 ^A^
	51–55	1.69 ^AB^	2.91 ^AB^	5.93 ^B^	18.73 ^AB^	2.63 ^AB^	30.08 ^AB^
	56–60	1.71 ^ABC^	2.88 ^ABC^	6.00 ^BCa^	18.66 ^BC^	2.64 ^ABC^	30.26 ^ABC^
	61–75	1.76 ^ABC^	2.76 ^ABC^	6.12 ^ABC^	18.32 ^ABC^	2.70 ^ABC^	30.61 ^ABC^
Lactation number	1	1.61 ^A^	2.83 ^A^	6.12 ^A^	18.49 ^A^	2.52 ^A^	28.17 ^A^
	2 or 3	1.79 ^A^	2.88 ^A^	5.88 ^A^	18.68 ^A^	2.76 ^A^	31.93 ^A^
Season of calving	Autumn	1.70 ^A^	2.90 ^A^	5.99	18.91 ^A^	2.63 ^A^	30.49 ^A^
	Spring	1.74 ^AB^	2.77 ^AB^	6.06 ^A^	18.11 ^AB^	2.69 ^AB^	29.76 ^AB^
	Summer	1.68 ^ABC^	2.85 ^BC^	5.99	18.57 ^ABC^	2.60 ^ABC^	29.47 ^ABC^
	Winter	1.70 ^BC^	2.91 ^BC^	5.97 ^A^	18.74 ^BC^	2.63 ^BC^	30.49 ^BC^
Age at first calving (months)	≤24	1.67 ^Aa^	2.76 ^A^	5.65 ^A^	18.43 ^A^	2.61 ^Aa^	28.03 ^A^
	[24–25)	1.59 ^Ba^	2.66 ^B^	6.33 ^A^	15.92 ^AB^	2.38 ^B^	25.42 ^AB^
	[25–26)	1.71 ^Bb^	2.90 ^ABC^	5.89	18.86 ^BC^	2.64 ^BCa^	30.73 ^ABC^
	[26–36)	1.76 ^AB^	3.08 ^ABCD^	5.95	21.61 ^ABCD^	2.75 ^AB^	34.92 ^ABCD^
	≥36	1.80 ^ABb^	2.88 ^ABD^	6.17 ^A^	18.09 ^BD^	2.82 ^ABC^	31.15 ^ABD^
Days in milk (days)	50	1.64 ^A^	3.00 ^A^	6.25 ^Aa^	21.25 ^A^	2.47 ^A^	32.63 ^A^
	51–100	1.70 ^AB^	3.06 ^AB^	6.30 ^Ba^	21.51 ^AB^	2.55 ^AB^	34.24 ^AB^
	101–150	1.72 ^ABC^	2.97 ^ABC^	6.05 ^ABC^	19.47 ^ABC^	2.65 ^ABC^	31.74 ^ABC^
	151–200	1.73 ^ABCD^	2.87 ^BCD^	5.83 ^ABCD^	17.91 ^ABCD^	2.71 ^ABCD^	29.63 ^ABCD^
	201–250	1.73 ^ABCE^	2.74 ^ABCDE^	5.77 ^ABCD^	16.53 ^ABCDE^	2.73 ^ABCDa^	27.49 ^ABCDE^
	251–305	1.71 ^ABCDE^	2.51 ^ABCDE^	5.79 ^ABC^	14.83 ^ABCDE^	2.72 ^ABCDa^	24.58 ^ABCDE^
Rear quarter to total quarter MY ratio (%)	34–50	1.67 ^A^	2.76 ^A^	5.98 ^A^	18.03 ^A^	2.61 ^A^	28.57 ^A^
	51–55	1.73 ^AB^	2.87 ^AB^	5.92 ^AB^	18.44 ^AB^	2.70 ^AB^	30.49 ^AB^
	56–60	1.73 ^AC^	2.91 ^ABC^	5.94 ^C^	18.76 ^ABC^	2.68 ^ABC^	30.89 ^ABC^
	61–73	1.67 ^BC^	2.89 ^ABC^	6.15 ^ABC^	19.11 ^ABC^	2.57 ^ABC^	30.26 ^ABC^

ME—milking efficiency (kg/min); MF—milking frequency (n); AT—attachment time (s); BT—box time (min); MS—milk speed (kg/min); MY—milk yield (kg); A-E—in columns, separately for each effect, values marked with the same letters vary significantly at *p* ≤ 0.01; a, b—in columns, separately for each effect, values marked with the same letters vary significantly at *p* ≤ 0.05.

**Table 4:** Pearson’s linear correlation between milking efficiency and number of cows per one robot, age at 1st calving, milking frequency, attachment time, box time, milk speed, milk yield and rear quarter to total quarter MY ratio.

Trait	Milking Efficiency	*p*-Value
No. of cows per robot (n)	−0.043	<0.0001
Age at 1st calving (days)	0.081	<0.0001
Milking frequency (n/day)	−0.079	0.5544
Attachment time (s)	−0.161	<0.0001
Box time (min/day)	−0.483	<0.0001
Milk speed (kg/min)	0.879	<0.0001
Milk yield (kg/day)	0.229	<0.0001
Rear quarter to total quarter MY ratio (%)	−0.020	<0.0001

**Table 5:** Number of division rules and importance of tested variables in tree creation based on Importance measure.

Variable	Number of Splits	Importance	Importance of Validation
Milk yield	4	1.000	1.000
Milking frequency	2	0.984	0.982
Attachment time	10	0.917	0.921
Days in milk	5	0.678	0.679
Lactation number	2	0.550	0.547
Number of cows per robot	1	0.531	0.529
Rear quarter to total quarter MY ratio	3	0.259	0.265
Age at 1st calving	1	0.131	0.125
Season of calving	1	0.104	0.102

## Data Availability

Data is contained within the article.
